# Effects of Immunization with Recombinant *Schistosoma mansoni* Enzymes AK and HGPRT: Murine Infection Control

**DOI:** 10.3390/pathogens12010069

**Published:** 2023-01-01

**Authors:** Ana Carolina Maragno Fattori, Elisandra de A. Montija, Bruna D. de L. Fragelli, Ricardo de O. Correia, Cynthia Aparecida de Castro, Larissa Romanello, Camila T. Nogueira, Silmara M. Allegretti, Edson G. Soares, Humberto D. Pereira, Fernanda de F. Anibal

**Affiliations:** 1 Laboratório de Inflamação e Doenças Infecciosas, Departamento de Morfologia e Patologia, Universidade Federal de São Carlos, São Carlos 13565-905, Brazil; 2 Departamento de Saúde e Psicologia, Universidade do Estado de Minas Gerais, Ituiutaba 38302-192, Brazil; 3 Departamento de Bioquímica, Escola Paulista de Medicina, Universidade Federal de São Paulo, São Paulo 04039-032, Brazil; 4 Instituto de Biologia, Departamento de Biologia Animal, Universidade Estadual de Campinas, Campinas 13083-970, Brazil; 5 Laboratório de Citopatologia, Departamento de Patologia e Medicina Legal, Universidade de São Paulo, Ribeirão Preto 14040-900, Brazil; 6 Instituto de Física de São Carlos, Universidade de São Paulo, São Carlos 13566-590, Brazil

**Keywords:** immunization, Adenosine Kinase, Hypoxanthine-Guanine Phosphoribosyltransferase, schistosomiasis mansoni

## Abstract

Schistosomiasis is one of the most important human helminthiases worldwide. Praziquantel is the current treatment, and no vaccine is available until the present. Thus, the presented study aimed to evaluate the immunization effects with recombinant *Schistosoma mansoni* enzymes: Adenosine Kinase (AK) and Hypoxanthine-Guanine Phosphoribosyltransferase (HGPRT), as well as a MIX of the two enzymes. Female Balb/c mice were immunized in three doses, and 15 days after the last immunization, animals were infected with *S. mansoni*. Our results showed that the group MIX presented a reduction in the eggs in feces by 30.74% and 29%, respectively, in the adult worms. The groups AK, HGPRT and MIX could produce IgG1 antibodies, and the groups AK and MIX produced IgE antibodies anti-enzymes and anti-*S. mansoni* total proteins. The groups AK, HGPRT and MIX induced a reduction in the eosinophils in the peritoneal cavity. Besides, the group AK showed a decrease in the number of hepatic granulomas (41.81%) and the eggs present in the liver (42.30%). Therefore, it suggests that immunization with these enzymes can contribute to schistosomiasis control, as well as help to modulate experimental infection inducing a reduction of physiopathology in the disease.

## 1. Introduction

Schistosomiasis is a disease that primarily affects socially and economically disadvantaged populations, found in 78 countries in which 290.8 million individuals required preventive treatment in 2018 and only 97.2 million received treatment [1]. The intravascular helminth trematode of the genus *Schistosoma* is the parasite that causes schistosomiasis, a disease with great potential for dissemination [2,3,4], and the species *Schistosoma mansoni*, endemic in Africa, the Antilles and South America, causes one of the intestinal schistosomiasis diseases [2,5,6].

The specific treatment of schistosomiasis is done through the drug Praziquantel, which is effective against all *Schistosoma* species, but only acts on mature adult worms [7,8,9]. In endemic regions, mass treatment is not very effective since the drug does not prevent reinfection, and it is necessary to repeat the treatment through periodic administration as the non-susceptible schistosomula gives rise to a new generation of adult worms one to two months after treatment [10]. In addition to these factors, chemotherapy does not reverse the pathology already installed in the body, which is important considering the difficulty in diagnosing the disease in its early stage due to non-specific symptoms [11]. Thus, it is clear the need to search for new alternatives to improve the treatment and schistosomiasis control, such as the use of vaccines and the development of effective new drugs [11,12].

Due to the complexity of the parasite cycle, a vaccine against schistosomiasis, even if not 100% effective, would contribute in a very relevant way to reduce infections and decrease or even interrupt the transmission of the disease [10]. There are only three antigens in clinical trials for schistosomiasis mansoni, Sm14, Sm-TSP-2 and Sm-p80, despite decades of studies. Furthermore, these antigens may not show protection in humans at the end of clinical studies, as observed with the recombinant antigen Sh28GST for *S. haematobium*, which justifies the search for new vaccine targets [13].

Nucleotides can be synthesized in living beings in two ways: either by the de novo pathway, which uses as metabolic precursors amino acids, ribose-5-phosphate, CO_2_ and NH_3_, or by the salvage pathway, which uses free bases and nucleosides released from the breakdown of nucleic acids, thus requiring less energy for the synthesis of the purine bases [14,15]. The purine salvage pathway was first studied in *S. mansoni* in the 1970s and 1980s by Senft and colleagues and Dovey and colleagues. These works demonstrated that the parasite does not have the de novo purine synthesis pathway and, therefore, the purine salvage pathway is used exclusively to provide these bases [16,17]. As a result, the proteins in this pathway may represent research targets to produce new vaccines.

One of the central enzymes of the purine pathway is the Adenosine Kinase (AK), which functions to catalyze adenosine phosphorylation to adenosine monophosphate (AMP) using adenosine triphosphate (ATP) or guanosine triphosphate (GTP) as phosphate donors [18]. Hypoxanthine-Guanine Phosphoribosyltransferase (HGPRT), another key enzyme of the purine pathway, catalyzes the reversible phosphoribosylation of hypoxanthine and guanine to inosine monophosphate (IMP) or guanosine monophosphate (GMP), respectively, and pyrophosphate, having 5-phosphoribosyl 1-diphosphate (PRPP) as a phosphate and ribose donor [19,20].

Intracellular proteins associated with microtube function have been studied as possible vaccine candidates [21,22] as well as housekeeping proteins despite their evident intracellular origin [23]. These studies originate from the fact that the parasite presents blind-ending gut and, thus, digestion in it occurs extracellularly and the residues are expelled into the bloodstream of the host [23]. These molecules expelled by the parasite interact with specific antibodies and other effectors of the host immune system and represent a set of vaccine targets to be researched [24,25]. Several enzymes of the life cycle were identified in *S. mansoni* extracts, among them phosphoribosyltransferases, kinases, diphosphokinases, desaminases and phosphorylases [26,27].

In this way, the present work proposed to evaluate the effects of immunization with the recombinant *S. mansoni* enzymes Adenosine Kinase (AK) and Hypoxanthine-Guanine Phosphoribosyltransferase (HGPRT) in addition to MIX with the two enzymes in the control of the experimental murine infection to contribute to the search for a candidate vaccine for schistosomiasis.

## 2. Material and Methods

### 2.1. Mice

Balb/c female mice weighing 15–18 g and 4 weeks of age were obtained from the animal facilities of the Centro Multidisciplinar para Investigação Biológica na Área da Ciência em Animais de Laboratório da Universidade Estadual de Campinas (CEMIB–UNICAMP), Brazil, and were maintained under suitable living conditions. All the protocols involving animal use in this study were licensed by the Ethics Committee of Animal Use (CEUA) of Universidade Federal de São Carlos, under license number 3-024/2014.

### 2.2. Recombinant Enzymes AK and HGPRT

The recombinant *Schistosoma mansoni* enzymes Adenosine Kinase (AK2 Smp_008360) and Hypoxanthine-Guanine Phosphoribosyl Transferase (HGPRT Smp_103560) were obtained by heterologous expression in recombinant *Escherichia coli* and purified in the Laboratório de Biologia Estrutural of Instituto de Física de São Carlos (IFSC), Universidade de São Paulo (USP) as previously described [28,29].

### 2.3. Immunization Protocol

Mice were randomly divided into five groups (six animals per group), and two independent experiments were performed. The immunization protocol consisted of three doses of the vaccine injected intraperitoneally in a 15-day interval regimen. In the first dose, mice were immunized with 100 µg of enzyme emulsified in 100 µg of Aluminium Hydroxide adjuvant and solubilized in sterile phosphate-buffered saline (PBS) 1X, totalizing 200 µL of solution per animal (Group AK: 100 µg of AK2; Group HGPRT: 100 µg of HGPRT Smp_103560; Group MIX (AK + HGPRT): 50 µg of AK2 and 50 µg of HGPRT Smp_103560).

In the subsequent boosters, mice were immunized with 50 µg of the enzyme emulsified in 50 µg of Aluminium Hydroxide adjuvant and solubilized in sterile PBS 1X, totalizing 200 µL of solution per animal (Group AK: 50 µg of AK2; Group HGPRT: 50 µg of HGPRT Smp_103560; Group MIX: 25 µg of AK2 and 25 µg of HGPRT Smp_103560). The control groups (control—without immunization and infection; infected—without immunization and infected, INF) received Aluminium Hydroxide adjuvant intraperitoneally, in the same dosage than the immunized groups, solubilized in sterile PBS 1X, totalizing 200 µL of solution per animal.

### 2.4. Parasite and Challenge

Fifteen days after the last booster, animals were challenged through infection with cercariae of *Schistosoma mansoni*. The life cycle of *S. mansoni* (BH strain from Belo Horizonte—MG, Brazil) was maintained in *Biomphalaria glabrata*, which is the intermediate host, at the Departamento de Biologia Animal, IB, UNICAMP. As the definitive host, Balb/c female mice were infected (Groups: Infected, AK, HGPRT and MIX) by means of exposure to a suspension containing 80 cercariae per animal using the tail immersion technique as described by Olivier and Stirewalt (1952) [30].

### 2.5. Parasitological Parameters

The counting of the eggs excreted in the feces was performed on the 47th day after infection. The feces were collected before the euthanasia were analyzed utilizing the Kato–Katz quantitative method [31] and the number of eggs/g feces was determined (HelmTest BioManguinhos FIOCRUZ). On the 48th day after infection, worm recovery was made by perfusion of hepatic and portal mesenteric vessels [32], and the percentages of reduction (eggs/g of feces and worms) were calculated according to Delgado et al. (1992) [33] using the formulation:

(1) $$\%\;\mathrm{worm}\;\mathrm{reduction}=\frac{\mathrm{Total}\;\mathrm{worms}\;\mathrm{infected}\;\left(\mathrm{control}\;\mathrm{group}\right)-\mathrm{Total}\;\mathrm{worms}\;\mathrm{immunized}\;\mathrm{group}}{\mathrm{Total}\;\mathrm{worms}\;\mathrm{infected}\;\left(\mathrm{control}\;\mathrm{group}\right)}\times 100$$

**Figure 1:**
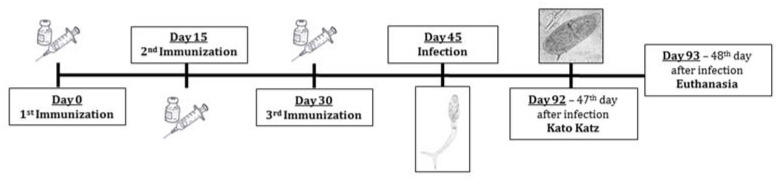
Immunization protocol and experimental design.

The immunization protocol and experimental design are schematized in Figure 1.

### 2.6. Immunological Parameters

Blood samples were obtained through the left brachial vein puncture using EDTA (0.3 M) as an anticoagulant, on the 48th day after infection, and plasma samples were obtained from blood after centrifugation at 1500 rpm for 15 min. Plasma levels of antibodies and cytokines were determined using Enzyme-Linked Immunosorbent Assays (ELISAs).

The extract containing the *Schistosoma mansoni* total proteins (STP) was obtained from about 150 worms of *S. mansoni* (BH strain), and protein extraction was performed by TRIzol^®^ reagent (Life Technologies, Carlsbad, CA, USA) according to the manufacturer’s instructions.

The ELISA for antibodies and cytokines detection was performed according to the manufacturer’s instructions using commercial kits (anti-Mouse IgG1 Antibody HRP Conjugated, Bethyl Laboratories, Inc. and OptEIATM, BD Biosciences for IgE, IL-4, IL-10 and IFN-γ). Briefly, Corning^®^ Costar 3590 96-well high-affinity microtiter plates were coated with 8 µg/mL of AK, HGPRT, MIX (AK + HGPRT) and STP in carbonate-bicarbonate buffer 0.1 M at pH 9.5 or with coating antibody (IL-4, IL-10 and IFN-γ) overnight at 4 °C.

Plates were washed with 300 µL/well of wash solution (phosphate-buffered saline, pH 7.4, with 0.05% Tween 20). Then, the plates were blocked with 200 µL/well of block solution (PBS, pH 7.4, plus 1% Bovine Serum Albumin—BSA) for 1 h at room temperature. The plates were washed again and incubated with 100 µL/well of the pool of plasma samples from each group diluted 1:10 in carbonate-bicarbonate buffer 0.1 M, pH 9.5 for antibodies and 100 µL/well of the pool of plasma samples from each group without dilution for cytokines at room temperature for 2 h.

After washing, the plates were incubated with HRP-conjugated anti-mouse IgG1 (Bethyl Laboratories, Inc.) and biotinylated anti-mouse IgE, IL-4, IL-10 and IFN-γ (BD Biosciences) and streptavidin-horseradish peroxidase-conjugated diluted according to the manufacturer’s instructions for 1 h and 30 min at room temperature. The color reaction was developed by Tetramethylbenzidine (TMB) incubation (BD Biosciences) under light and stopped with stop solution (sulfuric acid 2N). The plates were read at 450 nm in an ELISA plate reader (Thermoplate, microplate reader).

### 2.7. Eosinophils—Blood and Peritoneal Cavity

Peritoneal cavity cells were collected after the injection of 3 mL PBS containing 0.5% sodium citrate in the animal’s peritoneum. Counts for the total number of leukocytes in the blood and peritoneal cavity lavage (PCL) were performed using a Neubauer chamber with Turk solution for 1:20 dilution (Stibbe et al., 1985), and differential counts for blood were done on blood smear slides, and for PCL, they were obtained from slides prepared using cytospin (Serocito Mod. 2400 Fanem; 1500 rpm for 3 min) and stained with Rapid Panoptic (LB, Laborclin).

### 2.8. Histology

The liver of the mice was removed on the 48th day after infection and fixed in 10% buffered formaldehyde in PBS. Histological sections were produced using microtone and the slides were stained with Hematoxylin and Eosin and Masson trichrome for examination under light microscopy. Eggs and granulomas were counted in the slides of the liver, and the percentages of reduction was calculated according to Delgado et al. (1992) [33]. Slides were scanned in the Panoramic Desk (3D Histech) and the images were made in the Panoramic Viewer 1.15.4 (3D Histech) program.

### 2.9. Statistical Analysis

Data are presented as mean ± SD for each experimental group from two independent experiments (n = 12). Statistical analyses were performed using GraphPad Prism Inc. version 7 software. Normality tests were determined with the Shapiro–Wilk or Kolmogorov–Smirnov test according to the n. Analyses of the parametric data were performed by one-way ANOVA and were applied followed by the Tukey multiple comparisons test. Analyses of the non-parametric data were performed by Kruskal–Wallis tests followed by Dunn’s post-test. A value of *p* ˂ 0.05 was considered statistically significant.

## 3. Results

### 3.1. Immunization with a Combination of AK and HGPRT (MIX) Reduced the Number of Eggs in the Feces by 30.74% and Reduced the Number of Recovered Worms of the Animals by 29.00%

In Table 1 are represented the means ± SD from the number of eggs per gram of feces and the number of adult worms recovered of the animals from the experimental groups INF, AK, HGPRT and MIX (AK + HGPRT), as well as their respective percentages (%) of reduction of eggs in feces and adult worms.

The immunizations with the recombinant *S. mansoni* enzymes and subsequent infection with cercariae resulted in a decrease in the number of eggs eliminated in feces of the animals from the groups HGPRT and MIX when compared to the infected group. Among these immunized groups, the group that presented the highest percentage of egg reduction in the feces was the MIX group, with 30.74% of reduction (Table 1). This reduction contributes to the control of the biological cycle of the parasite and therefore the transmission of the disease.

Besides, the immunizations with the recombinant *S. mansoni* enzymes and subsequent infection with cercariae of the parasite resulted in a decrease in the number of adult worms recovered from the animals of the groups AK, HGPRT and MIX when compared to the INF group. Among the immunized groups, the group that presented the highest percentage of worm burden reduction was the MIX group, with a 29% reduction (Table 1).

### 3.2. Immunization with AK, HGPRT and MIX Induce Production of IgG1 and IgE Antibodies

**Figure 2:**
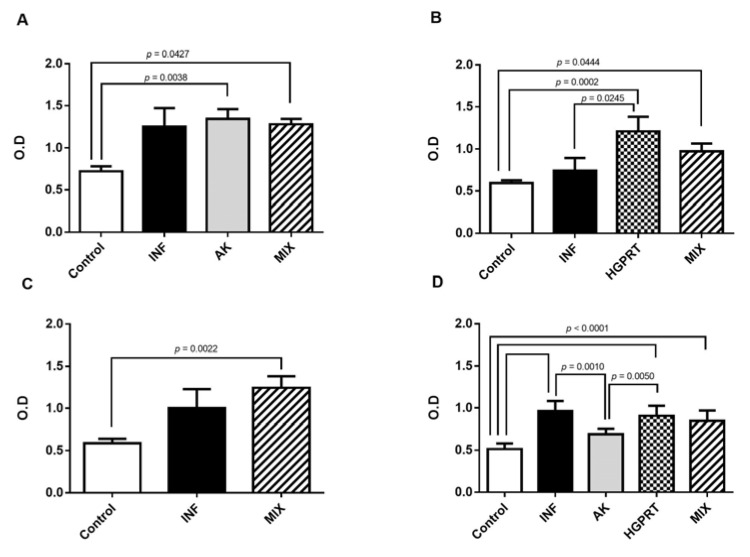
Detection of IgG1 antibody in the pool of the mice’s plasma on the 48th day after infection coated with the 8 µg/well of AK enzyme (**A**), HGPRT enzyme (**B**), MIX (AK and HGPRT) enzymes (**C**) and STP (*Schistosoma mansoni* total proteins) (**D**). Cumulative data are shown with two independent experiments and a total of n = 10–12 mice per group. Bar graphs with mean ± SD. For parametric data we used the one-way ANOVA test and Tukey’s post-test, and for non-parametric data, we used the Kruskal–Wallis test and Dunn’s post-test.

Figure 2 represents the means ± SD from the IgG1 antibody detection in the pool of the mice’s plasma from the experimental groups coated with AK enzyme (2A), HGPRT enzyme (2B), MIX enzymes (2C) and STP (2D). The immunization with the recombinant enzymes of *S. mansoni* was able to stimulate the production of IgG1 antibodies when the well was coated with AK, HGPRT and MIX enzymes and with STP. The group AK presented the highest O.D (Figure 2A) when compared to the other immunized groups (Figure 2B,C), and the group HGPRT showed a significantly higher O.D when compared to the INF group (Figure 2B).

**Figure 3:**
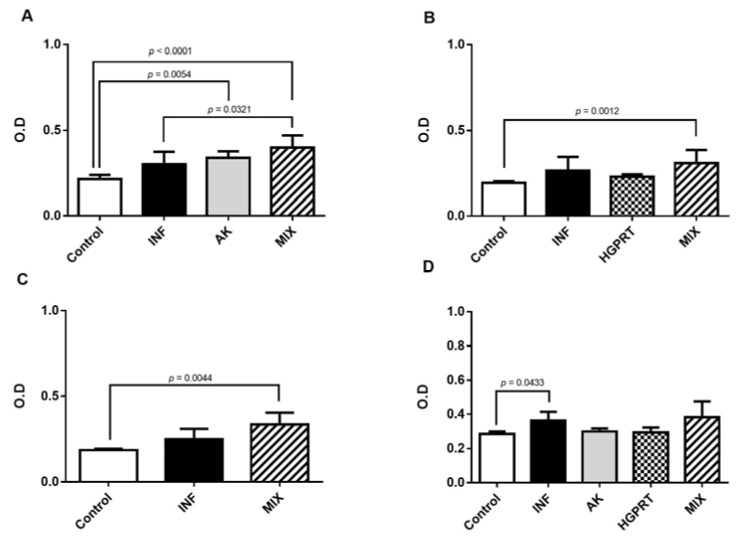
Detection of IgE antibody in the pool of the mice’s plasma on the 48th day after infection coated with the 8 µg/well of AK enzyme (**A**), HGPRT enzyme (**B**), MIX (AK and HGPRT) enzymes (**C**) and STP (*Schistosoma mansoni* total proteins) (**D**). Cumulative data are from two independent experiments and a total of n = 10–12 mice per group. Bar graphs with mean ± SD. For parametric data, we used one-way ANOVA test and Tukey’s post-test, and for non-parametric data, we used the Kruskal–Wallis test and Dunn’s post-test.

Figure 3 shows the means ± SD from the IgE antibody detection in the pool of the mice’s plasma from the experimental groups coated with the AK enzyme (3A), HGPRT enzyme (3B), MIX enzymes (3C) and STP (3D). The immunization with the AK and MIX recombinant *S. mansoni* enzymes was able to stimulate the production of IgE antibodies when the well was coated with AK, HGPRT and MIX enzymes (Figure 3A–C). The group MIX presented the highest O.D when compared with the group AK and showed a significantly higher O.D when compared to the INF group (Figure 3A).

### 3.3. AK, HGPRT and MIX: Cytokines and Eosinophils

**Figure 4:**
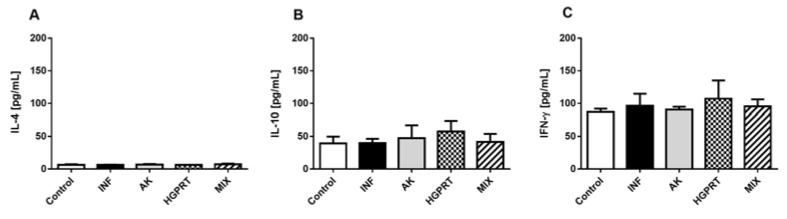
Level of detection for cytokines IL-4 (**A**), IL-10 (**B**) and IFN- γ (**C**), in pg/mL, in the pool of the mice’s plasma of the experimental groups on the 48th day after infection. Cumulative data are from two independent experiments and a total of n = 10–12 mice per group. Bar graphs with mean ± SD. There was no statistically significant difference among the groups analyzed for cytokines. For parametric data, we used the one-way ANOVA test and Tukey’s post-test, and for non-parametric data, we used the Kruskal–Wallis test and Dunn’s post-test.

Figure 4 shows the means ± SD from the IL-4 (4A), IL-10 (4B) and IFN- γ (4C) cytokines in the pool of the mice’s plasma of the experimental groups. There was no statistical difference among the groups in these cytokines. Despite this, the group HGPRT presented a higher mean IFN- γ concentration when compared to the other groups, suggesting a tendency for this cytokine to increase, which during the chronic phase of the disease, as is the case in our study, may be related to less collagen deposition and consequent fibrosis in the liver.

**Figure 5:**
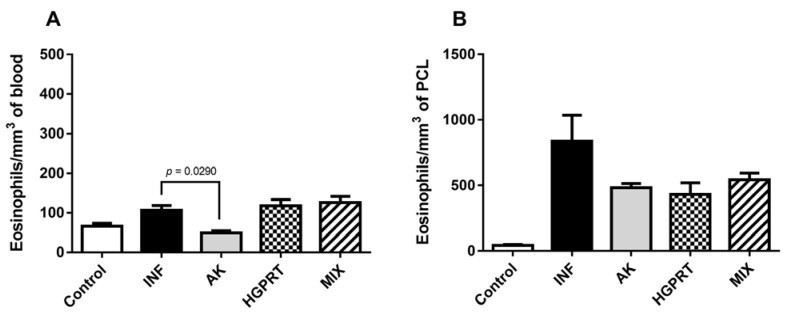
Quantification of eosinophils/mm^3^ in the blood (**A**) and in the Peritoneal Cavity Lavage (PCL) (**B**) of the experimental groups on the 48th day after infection. Cumulative data are from two independent experiments and a total of n = 10–12 mice per group. Bar graphs with mean ± SD. The statistical analysis was performed by the Kruskal–Wallis non-parametric test and Dunn’s post-test.

Figure 5 shows the means ± SD from eosinophils/mm^3^ in the mice’s blood (5A) and in the peritoneal cavity lavage (5B) of the experimental groups. In the blood (5A), the immunized group AK presents a reduction in the number of eosinophils when compared to the infected group. Even though the immunized groups (AK, HGPRT and MIX) did not present a statistical significance between the infected group, it is possible to observe a reduction in the eosinophils in the mice’s peritoneum (5B). This reduction is important due to the fact that eosinophils are one of the main cells that form the granuloma, which characterizes an important factor in the pathophysiology of schistosomiasis.

### 3.4. Liver Histology

**Figure 6:**
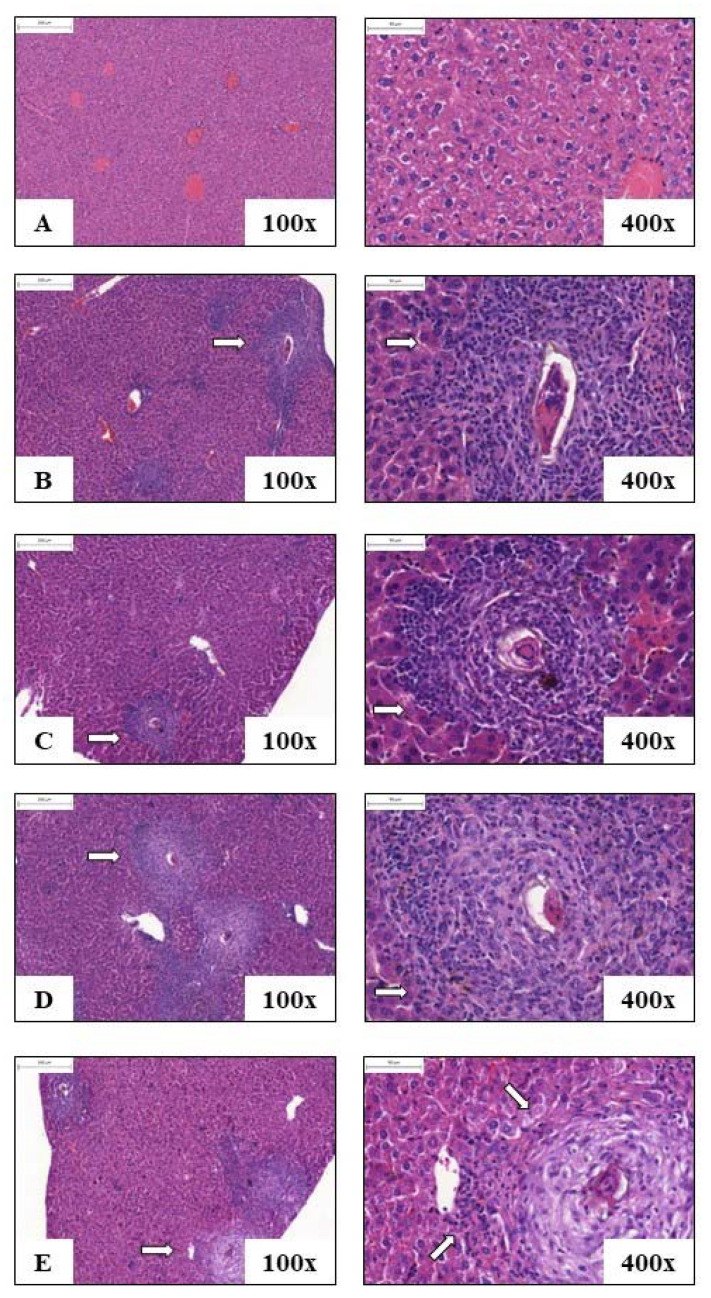
Representative histological cuts of liver from animals of each experimental group on the 48th day after infection stained with H&E: control (**A**), infected (**B**), AK (**C**), HGPRT (**D**) and MIX (**E**). The amplification is 100× (200 µm) and 400× (50 µm). Arrows indicate the periovascular granulomas and cellular infiltrate.

**Figure 7:**
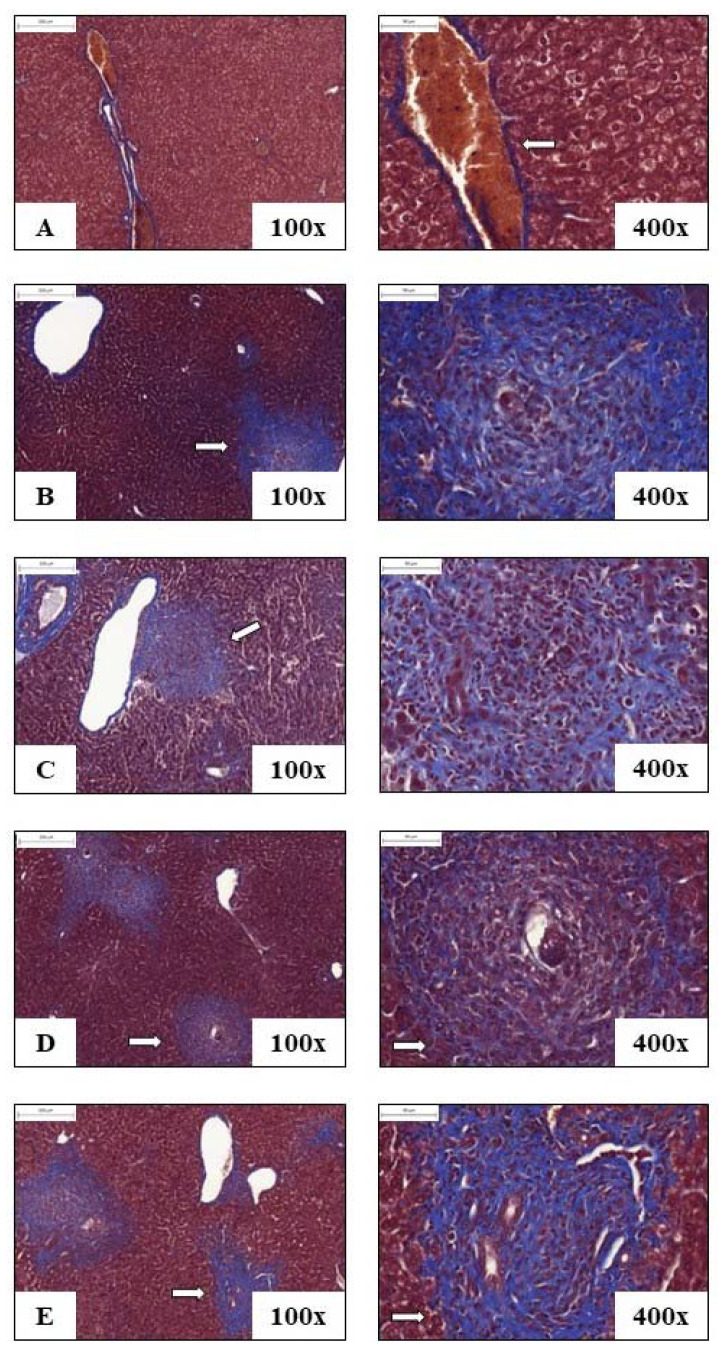
Representative histological cuts of liver from animals of each experimental group on the 48th day after infection stained with Masson’s Tricomic: control (**A**), infected (**B**), AK (**C**), HGPRT (**D**) and MIX (**E**). The amplification is 100× (200 µm) and 400× (50 µm). Arrows indicate the deposit of portal collagen and collagen deposits in periovascular granulomas.

Figure 6 and Figure 7 represent the photomicrographs of liver sections of animals from each experimental group: control, INF, AK, HGPRT and MIX. In Figure 5, the animals’ liver histological cuts were stained with H&E for analysis of the cells that make up the tissue and for observation of granulomas, and in Figure 6, the animals’ liver histological cuts were stained with Masson’s Tricomic for collagen deposition evaluation.

Mice of the control group showed preserved liver tissue without eggs deposited or granuloma formations (Figure 6A), and in the groups INF (6B), AK, HGPRT and MIX (6C, 6D and 6E), there was observed the periovascular granuloma formation, with mixed cellular infiltrate. In Figure 7, due to the staining with Masson’s Tricomic, a deposit of minimal portal collagen (in blue) can be seen in the liver of the control group (Figure 7A), while in the animals of the group INF (7B) and from the immunized groups AK, HGPRT and MIX (7C, 7D and 7E), the presence of collagen deposits in the periovascular granulomatous areas is observed.

Table 2 shows the means ± SD from the number of granulomas and eggs found in the histological sections of the animals’ liver in each experimental group: infected (INF), AK, HGPRT and MIX, as well as their percentages (%) of reduction. Among the immunized groups, the group that presented the reduction of granulomas and eggs in the liver was AK, with 41.81% of reduction for granulomas and 42.30% for eggs in the liver (Table 2).

## 4. Discussion

Schistosomiasis is considered one of the most important human helminthiases, affecting almost 240 million people worldwide. It is endemic in tropical and subtropical areas where more than 700 million people live. Limited options are available for the treatment, and Praziquantel is the drug used according to the World Health Organization as a disease control strategy [1]. However, this drug does not prevent constant reinfections in endemic areas and is not able to reverse the pathology already installed in the body, which contributes to the high morbidity of the disease [10,11].

Due to the fact that the parasite cycle is complex, the control and prevention of schistosomiasis requires an integrated and diversified approach, and developing a vaccine is presented as a key element [10]. Even after decades of studies searching for possible antigens for a vaccine against the disease, currently, there are only three such antigens in clinical studies for schistosomiasis mansoni (Sm14, Sm-TSP-2 and Sm-p80), which demonstrates the need for studies that will help to better understand the disease [13]. The use of recombinant enzymes involved in the metabolism of parasite purines, for the immunization of Balb/c mice and their subsequent challenge through *S. mansoni* infection, aimed to evaluate the effects of these enzymes on the control of experimental schistosomiasis mansoni to add new knowledge about the infection and the immunology of the disease.

The adult worm of the parasite does not reproduce within the final host, so a vaccine that can reduce the parasite load brings a huge benefit in terms of controlling the disease [13]. In addition, much of the installed pathology is due to the deposition of the parasite’s eggs in tissues, mainly in the liver, causing inflammatory process and fibrosis [34]. The results of our study show that the group immunized with MIX enzymes (AK+HGPRT) and later challenged with *S. mansoni* cercariae showed 30.74% reduction in the number of eggs in animal feces and 29% reduction in the number of adult worms when compared with the infected group.

This reduction in the number of eggs is quite relevant if it is taken into account that the immunization performed in this study used intracellular recombinant enzymes, when compared with other studies that showed a 59 to 60% reduction in the number of eggs eliminated in the feces from animals that were immunized with total proteins from the *S. mansoni* tegument [35], and from 52 to 60% of the number of eggs disposed of in the feces of animals immunized with an insoluble recombinant protein expressed in the gastroderm and tegument of adult *S. mansoni* worms [36]. In addition, another study from our research group showed a significant reduction in the number of eggs in the feces after immunization with the enzyme HGPRT, which is one of the enzymes that compose the MIX in this study [37].

Regarding the reduction in parasite load (29% in the MIX group), the value found in this study is higher than the value obtained with immunization with an insoluble recombinant protein expressed in the gastroderm and tegument of adult *S. mansoni* worms, which showed a reduction in parasite load from 16.21 to 25% [36]. The reduction of worm burden obtained from the immunization with MIX of recombinant enzymes is also very close to the values obtained in several other studies that used different *S. mansoni* proteins in the immunization in murine models and in which the recovery of worms was carried out between the 45th and 50th day of infection. Among these studies is the model that used the Sm14 immunization (a component of the authorized vaccine for clinical trials in Brazil since 2012) and subsequent infection with 100 cercariae, which showed a reduction of 36.9 to 49.5% in parasite load [38]. Additionally, the immunization with the Sm-p80 of *S. mansoni* (phase 1 of clinical tests initiated) and subsequent infection with 150 cercariae showed 46.87% reduction in parasite burden [13,39] and 27% reduction after immunization with the enzyme HGPRT present in the MIX of enzymes of this study [37].

Both immunological mechanisms, cellular and humoral, are fundamental for the effective control of *S. mansoni* infection [40]. The results of this study demonstrated significant levels of IgG1 antibody production after immunization with the recombinant *S. mansoni* enzymes AK, HGPRT and MIX. This showed the ability of these enzymes to induce specific immunity in animals used in the schistosomiasis murine model. Furthermore, the fact that the plasma of the immunized animals recognized the *S. mansoni* total proteins (STP) proved that the antibodies produced from the immunization with the recombinant *S. mansoni* enzymes AK, HGPRT and MIX are capable to recognize and bind to adult worms, potentiating the control of this infection by paths not yet clarified for this model.

Activation of the immune response during infection may favor the control of the infectious agent. IgG1 and IgG3 antibodies are the most efficient in binding to monocyte, macrophage and natural killer (NK) cell receptors and in triggering the cytotoxic response [41]. Activated macrophages can rupture the schistosomal tegument and make the cytosolic enzyme exposed to the antibody binding that could mediate cytotoxicity or compromise the parasite’s metabolic activities by inhibiting the enzyme in question [41]. Plasma IgG1 levels indicate a Th2-type immune response, which is observed in the chronic phase of schistosomiasis. This response reflects the major damage to liver tissue and the inflammatory process present in the disease. Thus, to have immunogens capable of regulating these mechanisms and contributing to the balance of the Th1/Th2 immune response is of fundamental importance for the control of morbidity associated with the severity of schistosomiasis mansoni.

Gryseels and colleagues (2006) [5] related acquired resistance in individuals from endemic regions and re-infections during schistosomiasis mansoni in these areas with an IgE antibody-mediated response. In the results, significant levels of IgE antibody production could be observed after immunization with the *S. mansoni* recombinant enzymes AK and MIX. The immunization with a MIX of recombinant enzymes showed higher production of this antibody.

IgE antibodies activate mast cells in the skin and basophils in the blood, which are known to participate in immediate hypersensitivity reactions, a direct effector function in immunity against schistosomiasis [42]. This class of antibodies is also associated, in experimental models, with the reduction of the parasite load and decrease in the parasite fecundity [42], observed also by the immunization with MIX of recombinant enzymes in our work.

The presence of IgG1 indicates activation of the Th2 response, where mainly IL-5 and IL-4 are produced. IL-4 is known to be fundamental for the change of antibody class for IgE [43]. Regarding the dosage of IL-4 in the animals’ plasma, the results showed similar concentrations of this cytokine in the animals of the analyzed groups (control, infected, immunized AK, immunized HGPRT and immunized MIX). However, it is possible to notice a slight increase in the concentration of IL-4 in the groups immunized with AK and a MIX of recombinant enzymes. Thus, it is possible to suggest that the immunization was able to stimulate a Th2 response and indirectly increase levels of circulating IgE, which may favor resistance to the development of the infection in its most severe form.

The production of IL-10, a regulatory cytokine, plays an extremely important role in modulating the Th1 response and in the transition of the response type. In the chronic phase, there is the predominance of the Th2 response pattern, which plays a major role in the early stages of this phase. After this period, the Th2 response should be modulated to avoid the development of hepatic fibrosis and chronic morbidity [44]. From our results, it was observed that the concentration of IL-10 in the plasma of the animals that were immunized with recombinant *S. mansoni* enzymes (AK, HGPRT and MIX) showed a slight increase when compared to the control group and the infected group, although it is not statistically significant. As a regulatory cytokine of the adaptive immune response [45] and described as a “master” of immune regulation in the context of infectious diseases [46], it is important to have a balance in its concentration so that it does not present exacerbated values, neither for more nor for less, thus contributing to the regulation of immune response patterns in schistosomiasis.

IFN-γ is a Th1-standard cytokine that, along with IL-12, can protect against the IL-13-mediated fibrosis process in the chronic phase of schistosomiasis [44]. This cytokine participates in fibrolysis by inhibiting the multiplication of myofibroblasts and the production of extracellular matrix proteins [47]. In addition, studies in *S. mansoni* endemic regions have demonstrated the protective role of IFN-γ in the control of severe fibrosis [48]. From the results of our work, it can be observed that the concentration of IFN-γ in the plasma of animals immunized with the recombinant *S. mansoni* HGPRT enzyme presents a slight increase when compared with the infected group, although this difference is not statistically significant.

Given this fact, by staining with Masson’s Tricomic, it was possible to observe that the liver of the animals immunized with the recombinant *S. mansoni* HGPRT enzyme presented a trend towards less collagen deposition due to the blue staining appearing weaker in the representative histological sections of this group when compared to the other groups. This same group also showed a tendency to increase IFN-γ levels, a cytokine related to collagen deposition and fibrosis control, which is considered one of the most relevant aspects associated with the high morbidity of the disease. Thus, it is suggested that more quantitative studies of the liver should be performed in the future to clarify this trend.

The number of total leukocytes in the blood and body fluids, such as in the peritoneal cavity lavage, increases during infection due to the cellular response against the disease. However, after a certain period, these cells must be modulated, as their accumulation contributes to the formation of granulomas, which are composed, among others, of inflammatory cells of the immune system, with eosinophils being the most prominent cells since they represent 50% of the cells that form the granuloma [49,50].

Moreover, eosinophils also play a role in the post-inflammatory fibrotic process, in which these cells can stimulate the synthesis of collagen through the released cytokines and granules that interfere with the properties of fibroblasts [51]. Consequently, it is relevant for the control of the pathophysiology of the disease that there is the modulation of eosinophils during infection. Our results showed that eosinophils’ quantification in the blood of the immunized groups HGPRT and MIX is very similar to the infected group due to the stimulation of the infection, which suggests a characteristic eosinophilia in this type of helminthiasis. However, the group immunized with AK shows a negative regulation of this eosinophilia, and this same group showed a reduction of eggs and granulomas in the liver, suggesting a migration of these cells to the organ helping in the elimination of eggs and consequently in the reduction of granulomas. In the peritoneal cavity lavage, eosinophils show a trend towards downregulation by immunization of all enzymes evaluated in this study. This suggests immunomodulation by these at the site of infection.

The granuloma is composed of several inflammatory cells of the immune system (mainly eosinophils), extracellular matrix components, adhesion proteins, growth factors and angiogenesis, originating a spherical structure that surrounds each egg individually. After the death of the egg, the granuloma decreases, leaving fibrous plaques (with an abundant amount of collagen) in its place and increasing the portal blood pressure as well as the diameter of the portal vein [44,52]. Thus, it is relevant to note that the parasite egg is the main pathogenic agent, far outweighing the harmful effects produced directly by adult worms [53]. Our results showed that the group immunized with the recombinant *S. mansoni* AK enzyme had a reduction of 41.81% in the number of granulomas in the liver and 42.30% in the number of eggs in the liver when compared to the infected group.

Based on these findings, it can be suggested that the immunization with recombinant *S. mansoni* AK and HGPRT enzymes, as well as the MIX of these enzymes, presented antiparasitic activity, showing themselves as important immunogens for the construction of a vaccine against schistosomiasis. The MIX of enzymes presented itself as the best option in several parameters analyzed, confirming the hypothesis that there is a synergism between the enzymes AK and HGPRT to better control the infection in general when compared to the use of enzymes separately.

These are promising results for the use of recombinant *S. mansoni* AK and HGPRT enzymes as vaccine targets to combat schistosomiasis mansoni, and a better understanding of the mechanisms by which these enzymes act to control the disease, as well as the modulation of the inflammatory process regulating both innate and humoral immunity, is needed in this experimental model. It should be emphasized that the fight against schistosomiasis does not only include the search for new drugs or antigens for the synthesis of a vaccine against the disease, but also control measures, implementation of basic sanitation and health education, as well as awareness, especially among people who live in endemic areas.

## Figures and Tables

**Table 1:** Number of eggs per gram of feces and adult worms recovered of the animals and reduction percentage on the 47th and 48th day after infection, respectively.

Groups	Eggs/g of Feces	Reduction (%)	Adult Worms	Reduction (%)
INF	106.6 ± 46.68	-	24.89 ± 11.7	-
AK	132.8 ± 52.79	-	23.64 ± 13.49	5.02
HGPRT	104.2 ± 40.16	2.25	24.55 ± 12.69	1.36
MIX	73.83 ± 29.78	30.74	17.67 ± 5.85	29.00

Data are presented as mean ± SD from two independent experiments (n = 12 animals). The statistical analysis was performed by the one-way ANOVA parametric test and Tukey’s post-test. There was no statistical difference between the results of the immunized groups when compared with the INF group.

**Table 2:** Number of granulomas and eggs in the liver; reduction percentage of granulomas and eggs on the 48th day after infection.

Groups	Granulomas	Reduction (%)	Eggs in the Liver	Reduction (%)
INF	13.75 ± 5.67	-	6.5 ± 1.29	-
AK	8 ± 0.00	41.81	3.75 ± 2.75	42.30
HGPRT	14.67 ± 1.52	-	9 ± 5.47	-
MIX	15.75 ± 4.5	-	9.75 ± 5.18	-

Data are presented as mean ± SD from two independents experiments (n = 4 animals—representative). For parametric data, we used one-way ANOVA test and Tukey’s post-test, and for non-parametric data, we used the Kruskal–Wallis test and Dunn’s post-test. There was no statistical difference between the results of the immunized groups when compared with the INF group.

## Data Availability

Publicly available datasets were analyzed in this study. The data can be found here: https://drive.google.com/drive/folders/1VyXxdn9TOxKejRjkeKY_ZsqCyiaLug3v?usp=sharing (accessed on 8 November 2022).
